# Three-Dimensional Modeling of Weed Plants Using Low-Cost Photogrammetry

**DOI:** 10.3390/s18041077

**Published:** 2018-04-03

**Authors:** Dionisio Andújar, Mikel Calle, César Fernández-Quintanilla, Ángela Ribeiro, José Dorado

**Affiliations:** 1 Institute of Agricultural Sciences, CSIC, 28006 Madrid, Spain; cesar@ica.csic.es (C.F.-Q.); jose.dorado@csic.es (J.D.); 2 National Museum of Natural Sciences, CSIC, 28006 Madrid, Spain; m.calle@mncn.csic.es; 3 Centre for Automation and Robotics, CSIC-UPM, Arganda del Rey, 28500 Madrid, Spain; angela.ribeiro@csic.es

**Keywords:** plant phenotyping, RGB imagery, structure from motion, multi-view stereo, digital surface models

## Abstract

Sensing advances in plant phenotyping are of vital importance in basic and applied plant research. Plant phenotyping enables the modeling of complex shapes, which is useful, for example, in decision-making for agronomic management. In this sense, 3D processing algorithms for plant modeling is expanding rapidly with the emergence of new sensors and techniques designed to morphologically characterize. However, there are still some technical aspects to be improved, such as an accurate reconstruction of end-details. This study adapted low-cost techniques, Structure from Motion (SfM) and MultiView Stereo (MVS), to create 3D models for reconstructing plants of three weed species with contrasting shape and plant structures. Plant reconstruction was developed by applying SfM algorithms to an input set of digital images acquired sequentially following a track that was concentric and equidistant with respect to the plant axis and using three different angles, from a perpendicular to top view, which guaranteed the necessary overlap between images to obtain high precision 3D models. With this information, a dense point cloud was created using MVS, from which a 3D polygon mesh representing every plants’ shape and geometry was generated. These 3D models were validated with ground truth values (e.g., plant height, leaf area (LA) and plant dry biomass) using regression methods. The results showed, in general, a good consistency in the correlation equations between the estimated values in the models and the actual values measured in the weed plants. Indeed, 3D modeling using SfM algorithms proved to be a valuable methodology for weed phenotyping, since it accurately estimated the actual values of plant height and LA. Additionally, image processing using the SfM method was relatively fast. Consequently, our results indicate the potential of this budget system for plant reconstruction at high detail, which may be usable in several scenarios, including outdoor conditions. Future research should address other issues, such as the time-cost relationship and the need for detail in the different approaches.

## 1. Introduction

Plant phenotyping allows researchers to gather information about plant architecture, which is fundamental to improve plant characterization, selection and discrimination [1]. Plant models through phenotyping processes are useful for assessing growth, physiology, architecture, stress, yield and every development in the plant, which allows plant management to be more comprehensive [2]. Plant modeling can be used to characterize stress from biotic or abiotic factors, biomass production, weed discrimination, fruit characterization, yield, leaf traits, root morphology, and photosynthetic efficiency, among other factors. Traditionally these factors have been assessed by experts relying on visual scoring, which creates differences between expert opinions and is time-consuming. Thus, the goal of plant phenotyping is to measure plant characteristics accurately, avoiding appreciative differences from different judges. Plant phenotyping is able to measure complex shapes and, therefore, is useful in decision-making for plant selection, treatment or agronomical management [3]. However, increasing knowledge and expertise require technological developments in sensing devices and processing methods. Many of the current sensing techniques are based on two dimensional characteristics, such as hyperspectral or thermal imaging, which are highly dependent on angle and distance to the target plants. Currently, 3D modeling is being proposed for the morphological characterization of plants. Three-dimensional modeling is rapidly expanding and new techniques are becoming more attractive. These techniques include visible images [4], LiDAR (Light Detection and Ranging) [5], structured light [6], spectroscopy [7], and thermal images [8]. The most common technique is visible imaging based on sensitive sensors within the range of what is visible, due to its economic price and ease of operation. The obtained images, under controlled conditions, can be related to yield, nutrition stress, vigor, biomass or other related parameters.

Certainly, each of these sensors provide valuable information; however, new sensors and techniques have arisen. New technologies are able to create 3D models, which accurately characterize plant shape and morphology, extracting several parameters that help in breeding and agronomic programs. Far from a unique system, the caption of the third dimension can be acquired by many ways and principles. For instance, the use of cameras and scanners positioned at different distances and angles in relation to the target allow us to acquire data according to our interests. Indeed, a camera usually takes, in one shot, two dimensions in a matrix with values of X-Y pairs. Nevertheless, a depth camera captures regarding the distance to the focus for each X-Y pair. In the case of scanners, they provide a dimensional line per reading. Therefore, a displacement of the sensor can be used to generate a third dimension. Light detection and ranging (LiDAR) is among the most commonly used methods due to its’ robustness and reliability in relation to the accuracy and resolution of this sensor. It has been used in several applications, from leaf area (LA) characterization to weed discrimination [9]. The LiDAR sensor allows for scanning at high frequencies and large distances. However, the costs increase as the resolution increases. In addition, edge detection is poor. Other drawbacks of the LiDAR sensor are the need for calibration, as well as the need to displace the sensor along the plant to create a point cloud. The data resulting from the aforementioned sensors cannot detect leaf overlapping and depth and images are not of high enough quality. When using multiple-angle modes, both type of sensors could increase information of the real 3D scene and overlaps could be reconstructed. Thus, a real 3D point cloud with X-Y-Z coordinates could be created with additional information, such as color, depending on the sensor. Similarly, RGB-D cameras, such as Kinect v2, have been widely used for plant characterization in agriculture. Andújar et al. [10] proposed the use of depth cameras for the detection of weeds in maize fields, concluding that discrimination of weeds and crops can be performed with low-cost sensors in the same way as with expensive laser scanners. Consequently, new possibilities for sensors, different from those traditionally used in plant phenotyping, are opening up. Nevertheless, there are still certain aspects that need to be improved, such as the accurate reconstruction of end-details, as well as the loss of some information necessary for more detailed application. The sensor MultiSense S7 from Carnegie Robotics combines lasers, uses depth cameras and stereo vision to reach a good resolution, but with high acquisition costs. Thus, a budget and accurate reconstruction method is required in some scenarios. The use of stereo vision has proved to have lower cost and reaches higher definition levels of plant reconstruction. It works in the same manner as human vision, using two eyes to view a scene. This methodology involves the use of two or more cameras or a camera moving around the plant. There are, however, certain adjustments that should be fine-tuned. For instance, the distance from the camera to the target plant, which should be calculated based on the focal length of the camera, the overlap between photographs and the relative rotation of the plant in various images. Also, the algorithm used greatly affects the accuracy of the depth model and non-controlled illumination, which can affect the quality of the models. Indoors applications have shown their capability for plant reconstruction. Takizawa et al. [11] reconstructed plant models and extracted information of plant height, LA and shape. Ivanov et al. [12] characterized maize plants in field conditions using images acquired from different angles to describe plant structure, therefore using leaf orientation, LA distribution and leaf position. The combination of cameras and images in Structure from Motion (SfM) techniques usually produce a sparse set of 3D points. This methodology consists of estimating a set of points from a common position of cameras. From this set of points and by means of a region growing algorithm, a dense cloud of points is created [13]. Similarly, the Multi-View Stereo (MVS) technique allows for the creation of dense 3D models through the fusion of images, even in real time [14], and budget hardware. These techniques open up the possibility of creating plant models with a high level of detail. However, in order to reduce costs, the possibilities of using a single camera without any other additional equipment is still a challenge. The development of algorithms for image fusion and the quality of models needs to be explored. Thus, the main objective of this work was to assess the possibilities of low-cost techniques, such as the coupling of SfM and MVS to reconstruct and differentiate weed plants according to their classification in dicotyledonous (from now on, dicot) and monocotyledonous (from now on, monocot) groups. The weed species chosen for model reconstruction are among the most troublesome weeds in maize crops in Mediterranean regions. Specifically, we explored the capabilities and limitations of these modeling techniques to accurately extract various parameters related to the morphology of these two groups of weeds.

## 2. Materials and Methods

Three weed species with contrasting shape and plant structures were selected for image processing on a commercial maize field (Arganda del Rey, Madrid, Central Spain). Two dicots, *Xanthium strumarium* L. and *Datura ferox* L., and one monocot, *Sorghum halepense* L., were chosen for the experiment, looking for structural differences. These weed species were selected for their contribution to economic losses due to yield reduction in maize crops. Image acquisition was carried out during May 2017 when maize was at stage 12 to 14 of the BBCH (Biologische Bundesanstalt, Bundessortenamt und CHemische Industrie) scale [15] and weeds ranged from BBCH 10 to BBCH 20. At sampling time, weed height ranged from 4 cm to 30 cm. The samples were chosen for their different weed stages. A total of ten samples per weed species were monitored, which were selected randomly within the field looking for a representative sample of the population, that is, within a 95% confidence interval of mean plant height. Weeds were usually located between the maize lines, a crop planted in lines 75 cm apart. Our study focused on this area between crop lines, which in the early stages of growth are not covered by the crop. Consequently, the crop plants did not interfere in the model creation and only weeds were monitored. In addition, the adjacent weed plants were removed in order to avoid interference between monocots and dicots that could affect the model reconstruction of each group of weeds. Since the adjacent weed plants were often of the same species, the use of filters based on color could not discriminate and isolate the target weed, which would have affected the accuracy of the model. Three 10 cm graphic scales were located on the ground following a triangular shape around the plant, leaving the target plant in the center of the triangle. Graphic scales were useful not only for model creation but also for scaling the model to actual measurements in post-processing.

### 2.1. Image Acquisition, Field Measurements and Data Processing

**Figure 1:**
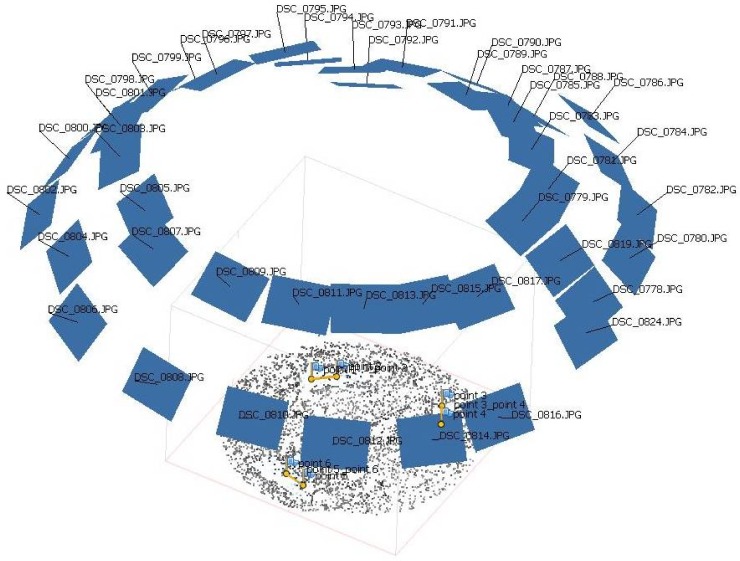
Example of a Set of images taken around a weed plant during image acquisition.

Plant reconstruction was achieved by applying SfM-MVS to an input set of images covering each individual weed. For this, the images were acquired sequentially following a concentric track with respect to the plant axis. Two rounds were done at different heights, thus obtaining two different oblique angles. A minimum of 20 images were taken at each track by orbiting the plant with a camera (Nikon D5500, Canon, Tokyo, Japan), shooting every ~15° to 18° of the circumference (see shooting scheme in Figure 1). The distance was kept constant at 50 cm to the plant from the camera objective. The rounds made during the image acquisition process resulted in a set of about 40 images which guaranteed a minimum overlap between the images of 90%. This overlap was necessary since the desired model reconstruction looked for high precision. A shadowing structure with a white photographic cloth was used during image acquisition in order to eliminate shadows and to avoid direct sun illumination while sampling. The separation between the shooting camera points was chosen according to the model resolution and the need for separation between points while moving the camera along the defined circular track. A full covering of weed plants required 40 to 50 images per plant according to its size. The camera positions were not predefined since variations were corrected by the algorithm used in the SfM-MVS technique for plant reconstruction. 

**Figure 2:**
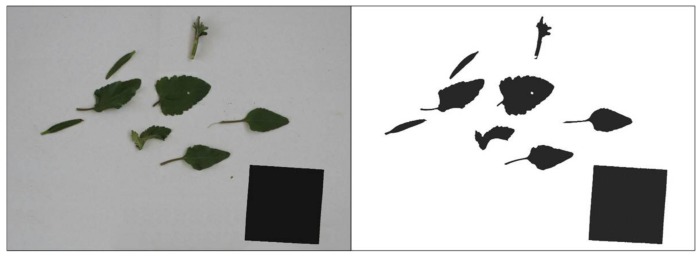
Example of RGB images (**left**) used to quantify LA, after their transformation to binary images (**right**) and subsequent application of Otsu’s thresholding method.

After acquiring the images, the actual height of every weed was manually measured. The use of height measurement in plant phenotyping is of high importance since the stem is an indicator of the growth response of plants. The actual plant height was measured using a cylinder extended from the base to the end of the main stem, thus indicating the maximum stem height [16]. Thereafter, weeds were taken to the laboratory for the determination of plant biomass and LA. Analyses of these parameters were important to determine the plant’s growing stage and health status. They were also indicators for operational tasks, such as herbicide applications [17]. The oven-dry weight of biomass was determined by drying the samples at 78 °C, over 48 h. For LA calculation, all leaves and stems were separated and placed on a white surface where an overhead digital image was acquired with a Nikon D5500 camera. A standard 100 cm^2^ black square was also placed in the image as a reference in order to calculate, by correlation, the LAI of each sample (Figure 2). The RGB images were transformed to binary images. A linear combination of the RGB planes with coefficients (R = −0.884, G = 1.262, B = −0.311) was performed. The applied coefficients were obtained by a genetic algorithm optimization process [18] that proved to perform better than Excess Green coefficients (ExG = 2G-R-B) [19]. In the processed grey level image, the green plants appeared bright, while the rest of the scene with a different color appeared dark. Following, the Otsu’s thresholding method [20], we separated the plant objects pixel-wise into foreground and background in a binary image. The binary image was processed to calculate the LA, which was determined by black pixels, while white ones denoted the background.

Previously, to build a plant solid 3D model, a dense point cloud was created using Agisoft PhotoScan^®^ Professional Edition software (Agisoft LLC, St. Petersburg, Russia) version 1.0.4, one of the most used SfM-MVS software in scientific research. PhotoScan is a photogrammetry program that processes images in a fully automatic way, with the exception of the manual location of metric rules used to scale the models and calibrate them. Calibration consisted in an assessment of the difference between the real distances observed on the metric rules and estimated values in the model. The creation of models included several phases: (1) aligning images; (2) building field geometry; and (3) dense point cloud generation. First, common points were detected and camera positions automatically solved. This facilitated the refinement of camera calibration parameters. Thereafter, the software searched for more points in the images to create a dense 3D point cloud, from which the final model was created. Point clouds were processed on an Intel CORE i7-4710HQ laptop with Windows 7, 16 GB of RAM memory and a 2 Gb Nvidia GeForce GTX graphic card with a Graphics Processing Unit (GPU).

**Figure 3:**
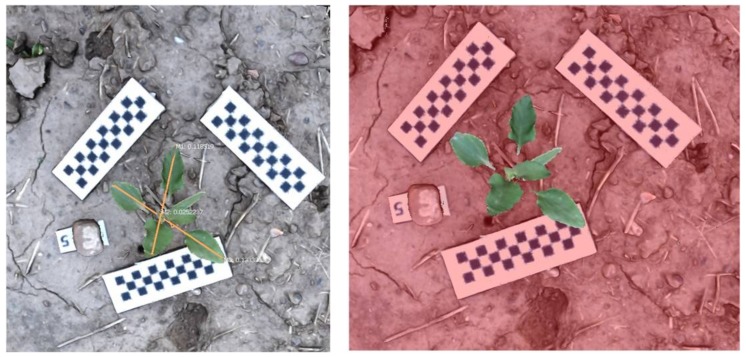
Model processing for distance and leaf area extraction.

Solid 3D models were processed off-line in the open source software Meshlab^®^ (Figure 3). This software was used to manage and plot the stored data, generating a model with the point cloud previously created in Agisoft. The software processes unstructured 3D models using remeshing tools and filters, which enables cleaning, managing, smoothing, and tools for the curvature analysis, visualization and processing of the created 3D models. The meshes were processed in different steps. (1) Filtering and removal of outliers and noise reduction by filter, where individual points were removed using statistical filters and points out of the grid by more than 0.5 cm were automatically removed. (2) Cleaning of neighboring weed plants, where plants were manually deleted from the point cloud. The target weed plant was isolated after removing every source of interference inside the bounding box. Although, the target plant was manually isolated by removing other plants, some leaves of nearby plants could interfere into the model due to their wide coverage. Some filters were tested to remove these parts. However, no filter was useful for isolating the target plant because of the high similarity in color and the continuous point cloud without significant space between the plants since the ground connected every plant into the model with a separation lower than 0.5 cm. Thus, the filter did not allow the automatic separation and a manual cleaning process was necessary. (3) Processing of the resulting meshes to extract the main parameters, which were compared with actual values. Plant height was estimated with Meshlab and compared with actual plant height. Similarly, leaf area (LA) was calculated from the triangulated 3D mesh by the sum of the areas of all triangles. The estimated LA was compared with actual LA, as well as dry biomass, for validating the models. 

### 2.2. Statistical Analyses

The 3D models generated were validated with the actual values using regression methods. Prior to the regression analysis, a correlation analysis was conducted to make an initial examination of bivariate relationships among the variables. Pearson’s correlation coefficient was used to analyze simple linear relationships between the estimated values generated by the model and actual ground truth values. The root mean square error (RMSE) and mean absolute percentage error (MAPE) indicators were calculated for error estimation. Both statistics provide an overall measure of how well the model fits the data.

## 3. Results and Discussion

**Figure 4:**
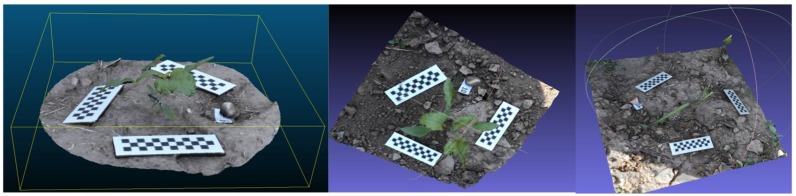
Examples of some different weed models.

In general, each individual plant was reconstructed three-dimensionally, with only small parts missing (Figure 4). For instance, some errors at the end of the leaves and branch borders occurred and some parts of the reconstruction did not show end-details, mainly in the monocot species *S. halepense*. The estimated parameters using the models showed a high correlation with the ground truth data. Indeed, a good consistency in linear correlation equations was found between the estimated parameters by the digital 3D model and the actual values measured in the weed plants. The parameters obtained in the models were highly correlated with the actual values, showing R^2^ from 0.998 to 0.832 and MAPE values were always under a tolerance limit of 4.57%. This limit error can be attributed to the error inherent in manual measurements [21]. In this sense, the level of detail can be improved and errors can be reduced by lowering the distance of the camera to the target plant and increasing the number of images per sample. Nevertheless, these modifications would be time consuming due to the increase in acquisition and processing times.

**Figure 5:**
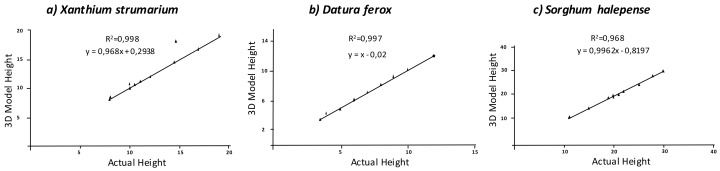
Regression analyses comparing actual weed height versus estimated height using 3D modeling.

Analysis of the correlation of estimated and measured plant heights using a cylindrical approach showed a good agreement between both measurements. The actual height of *X. strumarium* plants averaged 12 cm and fitted accurately to the model with R^2^ = 0.998 and a significance of *p* < 0.01. The high accuracy of the prediction was confirmed by a RMSE value of 0.043 cm and a MAPE value indicating a deviation of only 0.53% of the value estimated by the model in relation to the actual height value. In the case of the other dicotyledonous species, the actual height of *D. ferox* averaged 7.25 cm, a value that the 3D model always overestimated. The maximum height with a MAPE value indicated a deviation of only 0.22%. Nevertheless, regression analysis showed a strong correlation (R^2^ = 0.997) between the actual values and those extracted from the model (Figure 5). Comparison of the *D. ferox* model and ground truth values showed a RMSE of 0.018 cm. Thus, both dicots showed very similar results in terms of height estimation and there were no significant differences between models regarding species. In contrast, values of RMSE were higher in the monocot weed *S. halepense*, with a value of 0.878 cm and a MAPE of 4.57% of difference to the actual values. Despite this, the estimations were highly significant with R^2^ = 0.968. In the case of *S. halepense,* the use of a cylindrical approach to estimate stem length was more complicated than in the dicot weeds. The beginning and end of the stem were difficult to identify due to the insertion of leaves and stem curves. An automated recognition of the region of interest by image processing software could improve this result, for instance through color identification. The methodology would consist, initially, in a transformation from the RGB to the HSV. Each of the channels could be analyzed in terms of threshold separately with pre-defined intervals. From this segmentation, the region of interest defined by the stem could then be selected by a circular Hough transform applied to the binary image [9]. The application of this methodology in 3D models should be explored to increase accuracy in height determination. However, the current models had enough resolution for many of the weed phenotyping purposes. Similarly, RGB image discrimination algorithms can be adapted to 3D models to isolate individual plants from other plants in the scene in breeding programs. In agricultural scenarios, information would be extracted from a mix of weed plants for decision-making. Thus, individual plant information would not be required for weed management. Our results indicate the potential of this budget photogrammetry methodology for small weed reconstruction. Although, these results also point to a greater difficulty in reconstructing monocots with respect to dicots. Indeed, the elongated and thinner leave structure in monocots hinders the reconstruction of end-details. Similar results were obtained by Rose et al. [16], who created models at high resolution in lab conditions. Under these sampling conditions, illumination and wind were controlled to create high detailed models. However, they also showed some missing parts and triangulation errors at the leaf and branch borders.

**Figure 6:**
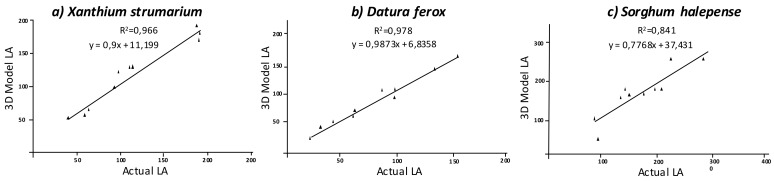
Regression analyses comparing actual LA and estimated LA using 3D modeling.

**Figure 7:**
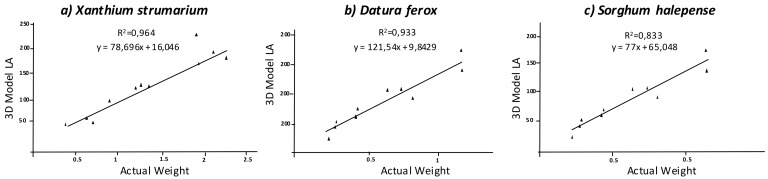
Regression analyses comparing estimated LA and actual dry biomass weight.

Similar to the above parameters, LA estimation using the 3D model was more accurate in dicots than in *S. halepense*, with very low differences between the former species. *Xanthium strumarium* plants showed an average actual LA of 116.34 cm^2^, while the value estimated by the model was 115.91 cm^2^. The correlation between both values was significant at *p* < 0.01 with R^2^ = 0.966 (Figure 6). The statistics showed high accuracy in model fit compared to actual LA measurements, with a RMSE value of 111.08 cm^2^ and MAPE value indicating a small negative deviation of −1.66%. Similarly, the model for *D. ferox* was properly fitted to ground truth with a RMSE value of 72.21 cm^2^, while the actual LA value averaged 78.63 cm^2^, which indicated a negative deviation of −10.55%, with respect to the actual value. That is, according to the regression analysis, both values were strongly correlated (R^2^ = 0.978). Although, both dicot species showed very similar results, the smaller size of *D. ferox* could lead to slightly more disperse results. In contrast, *S. halepense* species showed a lower accuracy of prediction than dicot species, despite being equally significant. Indeed, the regression analysis was significant at *p* < 0.01 with R^2^ = 0.841 (Figure 6). In addition, RMSE was relatively high, with a value of 569.80 cm^2^. However, the complex shape of this monocot species resulted in overestimation of the LA index in some samples while others were underestimated. This effect could be due to both leaf overlapping and leaf orientation, which creates big solid areas in those cases where the images did not cover empty areas. In other cases, leaf overlapping creates only a plane, which tends to underestimate LA. Increasing the number of images in more planes could cover these areas that are not overlapped, increasing the accuracy of the model. Due to this, the MAPE value showed a negative deviation of −2.59% in the model in relation to actual LA values. Comparison of the LA values estimated in the model and the actual values of dry biomass showed similar results (Figure 7), which confirm the good fit of the 3D model.

Therefore, considering these differences between species, 3D modeling using the SfM technique has proved to be an appropriate methodology for phenotyping and accurately estimating the actual values of height and LA. In addition, the SfM method is able to automatically calculate the position of the camera and avoid the use of scanning structures, as well as complex technologies for the acquisition of images such as stereo vision uses [22]. Indeed, since the camera position did not need to be fixed, the image acquisition process was relatively fast compared to other methods. Xiong et al. [23] established a high-throughput stereo-imaging system for 3D reconstruction of the canopy structure in oilseed rape seedlings. Although these authors proposed a more complex system, no differences were found to the results obtained in this work. In addition, image acquisition is faster than the cited studies and acquisition time can be improved by using more than one camera. In fact, they compared the estimated measurements using the model and the manual measurements, finding an absolute percentage error of automatic LA and plant height of 3.68 and 6.18, that is, similar values to those obtained by the SfM technique. Another study based on the phenotyping of monocot plants also resulted in a good reconstruction of maize plants [24]. However, they used simultaneous image acquisition to capture the stereo imagery pairs. Due to the complexity of the model, results only partially contained parts of the plants. Namely, stereo vision accuracy varied with the type of algorithm used and performance was adversely affected by the lack of surface texture of the target plant [2]. Indoors applications are accurate and helpful in breeding programs, allowing us to extract structural parameters such as LA or plant height from the created models. Mizuno et al. [25] developed an integral comprehensive tool capable of handling meteorological data, measuring and taking photographic images to analyze visualized information. They created a support system for farmers based on stereo vision that automatically supplies water when plants are close to wilting. Jin et al. [26] used indoor and outdoor stereo vision systems capable of detecting separated and overlapped corn plants with 96.7% success and errors ranging between 1 and 5 cm. Similar to our study, Santos et al. [27] used SfM but used indoor conditions and a fixed camera position with a tripod to reconstruct basil plants. These authors successfully described plant structure, even showing detailed leaf veins in the final model. However, they concluded that the method was not suitable for canopies that are too dense. On the other hand, Quan et al. [28] designed a rapid semi-automatic technique for modeling plants, employing the MVS technique for plant reconstruction. As in the current research, branch details were not fully reconstructed. However, the approximation to the real plant was good. In short, there are many studies aimed at developing methods for the reconstruction of plants simply, quickly and accurately. Although a high level of detail is sometimes required in breeding programs, the use of faster and simpler methods is usually more suitable for agronomical tasks. The use of SfM in outdoor conditions would allow the creation of detailed models with lower effort than other complex techniques. In addition, the use of this methodology could contribute to decision-making during plant growth under field conditions, which differ to plants cultivated under greenhouse conditions. However, some technical requirements should be adapted to future on-field applications. The use of on-field photogrammetric techniques would require automatic systems for image acquisition. The high number of images required for field modelling is a deterrent for it usage. The use of manual processes is suitable for research purposes. The integration of RBG cameras in autonomous platforms for field scouting would allow for the creation of high detail models of fields for decision-making processes. The integration of these methods on autonomous vehicles would also significantly reduce the cost by reducing human labor and it could integrate processes of machine vision, decision support and other applications in real time [29]. Although the current results were good enough in terms of model accuracy, this study focused only on single plants. Since plant leaf overlap can occur when weed density increases, real field conditions have to be tested to optimize the models for real agricultural tasks. 

Other outdoor technologies have shown their reconstruction capabilities. However, the level of detail is lower. Firstly, scanners such as LiDAR create a slide of points in 2D, but when the sensor is displaced to record the data, a 3D model can be reconstructed. Thus, the method is fast, and a high dense point cloud can be obtained independently of ambient light conditions. Although these advantages mean LiDAR methodology is widely used, the poor resolution does not properly detect leave edges or thin stems. On the other hand, there are other time-consuming aspects related to the need for calibration, as well as warming-up time. The lack of color information is sometimes a deterrent for it usage in some on field applications. Combination with image processing systems can create high detailed models. This approach has shown promise and commercial applications have arisen (e.g., FieldScan, Phenospex). Even though the approach is valid for breeding programs, the use of LiDAR in on-field applications, such as weed characterization, is sometimes limited to the use of plant height discrimination [30]. There has been recent advances in the use of Time of Flight (ToF) cameras, such as the Kinect device. This system has some advantages related to its low cost and the high detail obtained in the models aimed at plant phenotyping. Using the Kinect, Azzari et al. [31] and Andújar et al. [32] properly reconstructed vegetal structures under indoor and outdoor conditions. However, these cameras failed when reconstruction was to a high detail level. Sensor fusion has been proposed to increase the level of detail and reduce processing time. For instance, the combination of ToF cameras and imaging systems such as stereo vision would improve the level of detail in the model while constructing denser depth maps [33]. Consequently, plant phenotyping based on several sensors seems to be a promising methodology; however, the correct relationship between cost and efficiency should be established for each specific objective.

## 4. Conclusions

Plant phenotyping is possible through SfM and MVS technologies. Indeed, results of this work showed that weed plant reconstruction at a high level of detail is possible with a budget and simple system usable in several scenarios, even in outdoor conditions. Nevertheless, further efforts should focus on extracting additional structural information, such as leaf inclination, overlapping and stem diameter, which could be useful for decision-support systems. Aspects such as the time-cost relationship and the need for details in the different approaches should be assessed. End-details could be necessary for breeding programs or botanical classification; however, the integration of these models in on-field precision agriculture treatments would require less details and lower processing times. The limits and implications of detectable details should be studied. In this study, the target plants had different structures which led to different resolutions, while the image acquisition and operational processing systems were the same. The complexity and shape of the plants would need to be classified for automated decision-making programs.
